# Cefazolin Loaded Oxidized Regenerated Cellulose/Polycaprolactone Bilayered Composite for Use as Potential Antibacterial Dural Substitute

**DOI:** 10.3390/polym14204449

**Published:** 2022-10-21

**Authors:** Arunnee Sanpakitwattana, Waraporn Suvannapruk, Sorayouth Chumnanvej, Ruedee Hemstapat, Jintamai Suwanprateeb

**Affiliations:** 1 Department of Pharmacology, Faculty of Science, Mahidol University, Bangkok 10400, Thailand; 2 Biofunctional Materials and Devices Research Group, National Metal and Materials Technology Center (MTEC), Pathum Thani 12120, Thailand; 3 Neurosurgical Unit, Surgery Department, Faculty of Medicine Ramathibodi Hospital, Bangkok 10400, Thailand

**Keywords:** synthetic dural substitute, drug release, antibiotics carrier, cefazolin

## Abstract

Oxidized regenerated cellulose/polycaprolactone bilayered composite (ORC/PCL bilayered composite) was investigated for use as an antibacterial dural substitute. Cefazolin at the concentrations of 25, 50, 75 and 100 mg/mL was loaded in the ORC/PCL bilayered composite. Microstructure, density, thickness, tensile properties, cefazolin loading content, cefazolin releasing profile and antibacterial activity against *S. aureus* were measured. It was seen that the change in concentration of cefazolin loading affected the microstructure of the composite on the rough side, but not on the dense or smooth side. Cefazolin loaded ORC/PCL bilayered composite showed greater densities, but lower thickness, compared to those of drug unloaded composite. Tensile modulus was found to be greater and increased with increasing cefazolin loading, but tensile strength and strain at break were lower compared to the drug unloaded composite. In vitro cefazolin release in artificial cerebrospinal fluid (aCSF) consisted of initial burst release on day 1, followed by a constant small release of cefazolin. The antibacterial activity was observed to last for up to 4 days depending on the cefazolin loading. All these results suggested that ORC/PCL bilayered composite could be modified to serve as an antibiotic carrier for potential use as an antibacterial synthetic dura mater.

## 1. Introduction

Dura mater is the outermost membrane of meninges which surrounds the brain and spinal cord to retain the cerebrospinal fluid (CSF) inside. During neurosurgical procedures, intracranial lesions might lead to partial dura removal or dural perforation and the restoration of the dura mater to regain a watertight dural closure should be performed. The solitary use of native dura for such reconstruction could struggle to achieve an adequate primary closure in certain situations and might result in post-operative complications, morbidity and even loss of life [1,2]. Several dural substitutes and dural sealants are commercially available and used in place of native dura mater for dural repair and closure restoration. Recently, a new bilayered knitted fabric reinforced composite (ORC/PCL bilayered composite) was developed for potential use as a dural substitute [3,4,5]. This composite membrane contained two different morphologies including a composite layer which incorporated a relatively faster resorption ORC that could in situ generate pores for tissue ingrowth and a dense nonporous layer of relatively slower resorption PCL on the opposite side which helped provide the load bearing and liquid leakage resistance [6,7,8]. This type of dural substitute also offered several advantages including low cost, simple production as well as similar mechanical properties to human dura mater.

In general, post-operative CNS infection such as meningitis and post-operative surgical site infections (SSIs) have been reported following craniotomy and neurosurgical procedures [8,9]. Several risk factors associated with these post-operative infections have been identified, including CSF leakage, prolonged operation procedure (>3 h), diabetes, implantation of prosthetic devices such as shunt and cranioplasty materials [9,10]. The predominant pathogens causing these infections are Gram-positive bacteria, particularly *Staphylococcus aureus* (*S. aureus*) and *coagulase*-*negative staphylococci* (*CoNS*). The incidence rates of post-operative surgical site infections (SSIs) have been reported to decrease when antibiotic prophylaxis strategies were implemented [11,12]. The ideal antimicrobial drugs for prophylaxis should be effective against the most common organisms found at the surgical sites without eliminating other causative organisms [9,13]. Cephalosporins are the most commonly used prophylactic antibiotics in different types of surgical procedure [14]. In particular, cefazolin, the first generation cephalosporin, has been considered as the prophylactic drug of choice for post-neurosurgical infections [11]. This is because it is most effective against Gram-positive bacteria such as *S. aureus* and *CoNS*, which are the major causes of the post-operative SSIs and post-craniotomy infections. Moreover, several advantages of cefazolin have been reported including a low toxicity and low cost [12,15]. Cefazolin is given parenterally via intramuscular or intravenous administration since its absorption from the GI tract is insufficient.

Localized drug delivery is one of the most effective strategies for delivering drugs to the specific targeted site, which led to more preferable therapeutic effects [16]. It has been reported that a localized delivery system showed superiority over the conventional systemic antibiotic delivery [17]. A higher dose reaching directly to the desired site, decreased systemic toxicity, reduced the bacterial resistance as well as improved patient compliance [17,18,19]. The ideal localized system should exhibit a burst release of a large amount of drug in the initial period and be followed by a continual therapeutic dose release to prevent latent infection [16,19]. Cefazolin has been experimentally loaded into several carriers including a PCL sponge pad or fibers [20,21], PCL/sodium-alginate [22], mesoporous silicon microparticles [23], hydroxyapatite [24] and gelatin nanofiber mats [25,26] for use as localized drug release systems. Initial burst release of cefazolin was typically seen and followed by a sustained release from 24 h to 32 days depending on the type of carrier.

Preventing the incidence of post-neurosurgical infection is clinically crucial during neurosurgical procedures. Since an implant was reported to be one of the risk factors for SSIs, the use of an antibacterial dural substitute with the localized antibiotics delivery would probably help in decreasing this risk. This would be used as an adjuvant or add-on treatment and not as a replacement for systemic antibiotics prophylaxis via an intravenous route. Previously, commercial collagen artificial dura mater was experimentally mixed with cefuroxime sodium, ceftriaxone sodium or norvancomycin aimed to provide sustained drug release directly to the brain surface and it overcame the problem of the blood–brain barrier that lowered drug concentrations in the cerebrospinal fluid compared to the venous blood [27]. However, no antibacterial activity was carried out and only a short release study for up to 72 h was performed, although the release time of 6–7 days was designed. Vancomycin soaked commercial crosslinked collagen based dural substitute was also investigated for possible use in infected and contaminated wounds. However, the study was brief and only colony counting and antibacterial activity at 24 h were measured [28]. Therefore, data of an antibiotic loaded dural substitute that can perform dual functions for dural restoration and local release of antibiotics in the prevention of surgical site infection are still limited and not exhaustively studied. 

It was hypothesized that ORC/PCL bilayered composite which was developed and already passed in vitro and in vivo studies [3,4,5] could be further modified to act as an antibiotic dural substitute. The characteristics and properties of the carriers are important factors which could influence the drug releasing behavior of the material and the incorporation of drug in the carriers would in turn affect the property of the materials. This study was carried out to gain the understanding and knowledge to further develop an antibacterial dural substitute, which has rarely been reported. In this study, cefazolin was chosen as a model antibiotic for investigation. Various concentrations of cefazolin were loaded into previously selected ORC/PCL bilayered composite (P20 formulation) and their effects on physical properties, mechanical properties, drug loading and releasing characteristics and antibacterial activity were investigated for potentially being used as an antibacterial dural substitute in dural closure application.

## 2. Materials and Methods

### 2.1. Raw Materials

All raw materials including polycaprolactone (PCL, M_w_ ~ 80,000, Sigma-Aldrich, St. Louis, MI, USA), oxidized regenerated cellulose (ORC, Surgicel^®®^, Nu-Knit^®®^, Ethicon Inc., Raritan, NJ, USA) and N-methyl-2-pyrrolidone (NMP, Pharmasolve^TM^, Ashland Inc., Wilmington, DE, USA) were purchased and used in the as-received form. The antibiotic used was cefazolin sodium (Fazolin^®®^, Siam Bheasach Co., Ltd., Bangkok, Thailand) and was supplied in the powder form.

### 2.2. Sample Preparation

NMP was equally divided into two parts. Cefazolin powders were dissolved in the first part of NMP at the concentrations of 0, 25, 50, 75 and 100 mg/mL and stirred at room temperature until clear solution was obtained while 20 g of PCL was dissolved in the second part of NMP by heating at 60 °C using a hotplate until obtaining a clear viscous PCL solution. After cooling down to a temperature of approximately 40 °C, both parts were then mixed well together, producing 20% *w*/*v* cefazolin loaded PCL solutions. PCL solutions was used to evenly infiltrate both sides of ORC knitted woven fabric (30 × 70 × 1.0 mm^3^) and was then additionally recoated on one side of the loaded fabric as described previously [3,4]. The infiltrated and coated fabrics were then submerged in deionized water for 30 s to solidify the PCL and to leach out the solvent and they were then further dried in the oven at 40 °C for 24 h to obtain cefazolin loaded ORC/PCL bilayered composite (designated P20, P20_25, P20_50, P20_75 and P20_100, respectively). Cefazolin loaded PCL was also fabricated for use as comparative samples. The cefazolin loaded PCL solutions, which were prepared in the same manner as described previously, were poured into a cavity of a mold (30 × 70 × 1.0 mm^3^). The mold was then submerged in deionized water for 30 s and dried in the oven at 40 °C for 24 h to obtain cefazolin loaded PCL (designated PCL, PCL_25, PCL_50, PCL_75 and PCL_100, respectively). The concentration of cefazolin solution used to infiltrate the ORC/PCL bilayered composite or mix in PCL in this study was selected based on the concentration of cefazolin solution used for typical intravenous or intramuscular injection which ranges from about 20 mg/mL to 250 mg/mL. Table 1 shows the formulations that were employed to prepare the samples.

### 2.3. Microstructure

Microstructure of the fabricated samples was observed by using a scanning electron microscope (JEOL JSM-5410) using an accelerating voltage of 20 kV. Prior to observation, the samples were gold sputter coated to improve the conductivity and prevent charging of the samples. 

### 2.4. Bulk Density

Weight of the sample and its dimensions (width, length and thickness) were measured by a precision balance (Sartorious) and a vernier caliper (Mitutoyo), respectively. The bulk density was determined by dividing the weight by the calculated volume.

### 2.5. Tensile Properties

Rectangular specimens (10 mm × 70 mm) were cut from the fabricated samples and were used for tensile testing following ISO 527-4 to determine tensile modulus, tensile strength and tensile strain at break. All the tests were performed by a universal testing machine (Instron 55R 4502) equipped with a 10 kN load cell using a constant crosshead speed of 50 mm/min at 23 °C and 50% RH. 

### 2.6. Total Cefazolin Loading Content

Specimens (10 mm × 10 mm) were immersed in a bottle containing 5 mL of artificial cerebrospinal fluid (aCSF; 6.279 g/L sodium chloride, 0.216 g/L potassium chloride, 0.353 g/L calcium chloride, 0.488 g/L magnesium chloride, 1.932 g/L sodium bicarbonate, 0.358 g/L disodium hydrogen phosphate and 2-[4-(2-hydroxyethyl)piperazin-1-yl] ethanesulfonic acid (HEPES) 11.915 g, pH 7.4 [27]) and incubated for 24 h at 37 °C in an incubator. The specimen was taken out and aCSF was collected and filtered. The absorbance of the collected aCSF was then measured by using a UV–Vis spectrophotometer (Perkin Elmer) at a wavelength of 270 nm and compared with a linear equation of the calibration curve (cefazolin in aCSF, R-squared = 0.9996) to quantify the amount of released cefazolin from the sample. The standard cefazolin solutions ranged from 0.02, 0.05, 0.10, 0.25, 0.5, 1, 5, 10, 15, 20, 25 to 30 μg/mL. The collected sample was further dried at 40 °C and dissolved in 5 mL dimethylformamide (DMF) in a shaking incubator for 24 h at 37 °C, 100 rpm. The DMF solution was then filtered through a silica glass filter and the amount of cefazolin was quantified by using a UV–Vis spectrophotometer in a similar manner as that for aCSF but using the linear calibration curve of cefazolin in DMF solutions (R-squared = 0.9867) instead. The total amount of cefazolin in the samples was calculated by combining the measured cefazolin amount in the aCSF and that in DMF solution. The measurement was performed in triplicate and the result was expressed as the ratio of the total amount of cefazolin (mg) to the mass (g) of each specimen.

### 2.7. In Vitro Cefazolin Release Study

Specimens (10 mm × 10 mm) were prepared and each sample was placed in a plastic bottle containing 5 mL of aCSF and secured in place with a frame sheet to ensure that both sides of the sample were totally immersed in the solution. This was then incubated in an incubator at 37 °C. Every 24 h, the specimen was removed, slightly blotted with tissue paper and placed in a new bottle containing 5 mL of fresh aCSF. The remaining solution was collected and filtered with a 0.45 μm syringe filter (Corning). The absorbance of cefazolin in the collected solution was then measured by using a UV–Vis spectrophotometer (Perkin Elmer) at a wavelength of 270 nm and compared with a linear equation of the prepared calibration curve (cefazolin in aCSF; R-squared = 0.9996) as previously described to quantify the amount of cefazolin released from the sample. 

### 2.8. Minimum Inhibitory Concentration (MIC)

The MIC value of cefazolin used against Gram-positive *S. aureus* ATCC 25923 was assessed by using the standard broth macrodilution method as described previously [29]. Cefazolin solution was prepared by reconstituting 1 g vial cefazolin with 10 mL sterile aCSF to give a resultant concentration of 100 mg/mL and further diluted to obtain a stock solution of 0.01 mg/mL (10 μg/mL). A series of 2-fold higher concentrations of cefazolin than the final dilution range as well as control was prepared in duplicate as follows: 10, 5.0, 2.5, 1.25, 0.625, 0.3125, 0.1560, 0.0780, 0.0390 and 0.000 μg/mL. In addition, uninoculated wells of antibiotic-free broth were also prepared to ensure the sterility of the cefazolin solution and aCSF. All the wells were then incubated at 35 °C ± 2 °C for 18–20 h. The MIC value was determined as the lowest dilution of cefazolin that completely inhibited the visible growth of the test organism which is evidenced by the absence of turbidity in the well. The bacterial inoculum was prepared according to a standard broth culture method (European Committee for Antimicrobial Susceptibility Testing of the European Society of Clinical and Infectious, 2000). In brief, the bacterial colonies were taken with a sterile loop and transferred to 5 mL of sterile nutrient broth (NB). The broth was then incubated in a shaking incubator at 37 °C until the visible turbidity (4–6 h) was observed. The density of the suspension of bacterial culture was diluted with NB to give a turbidity equivalent to the 0.5 McFarland standard (approximately 1.5 × 10^8^ cfu/mL). This was performed using visual inspection such that the appearance of black lines was compared when observed through the bacterial inoculum and McFarland standard suspension. This inoculum was then transferred to a tube, which was further adjusted with NB to achieve a final organism density of 1.5 × 10^6^ cfu/mL.

### 2.9. Antibacterial Activity

The antibacterial activity of the samples was evaluated by using the agar disk-diffusion method or Kirby–Bauer test. The *S. aureus* ATCC 25923 (1.5 × 10^6^ cfu/mL) were inoculated on Mueller–Hinton agar plates. Specimens (10 mm × 10 mm) were then gently placed down to ensure even contact to the agar surface. Cefazolin loaded ORC/PCL bilayered composites contained two distinct surfaces, namely rough or porous surface (R) and smooth or dense surface (S), resulting from the processing. Both surfaces of the composite were independently evaluated whereas only one surface of the cefazolin loaded PCL was tested due to indistinguishable appearance between the two sides. All the plates were then incubated at 37 °C for 24 h, in which the samples were transferred to fresh *S. aureus* agar plates every 24 h. This was performed repeatedly until the inhibition (clear) zones were not observed. The size of the clear space around the sample indicated the antibacterial activity of the sample. All dimensional measurement was carried out by using a vernier caliper and used to calculate the inhibition zone values using the following formula:

H = (D − d)/2,(1)

where H is the inhibition zone (mm);

D is the clear space diameter around the sample (mm);

d is the specimen diameter (mm).

### 2.10. Statistical Analysis

The data were described as the mean ± standard deviation values. Data were analyzed by using statistical analysis software (GraphPad Prism version 6, GraphPad Software, San Diego, CA, USA). One-way analysis of variance (ANOVA), followed by Tukey’s multiple comparisons test, was employed to determine the significant between groups or samples. A *p*-value < 0.05 was considered statistically significant.

## 3. Results

### 3.1. Physical and Mechanical Properties

#### 3.1.1. Microstructure, Bulk Density and Thickness

**Figure 1:**
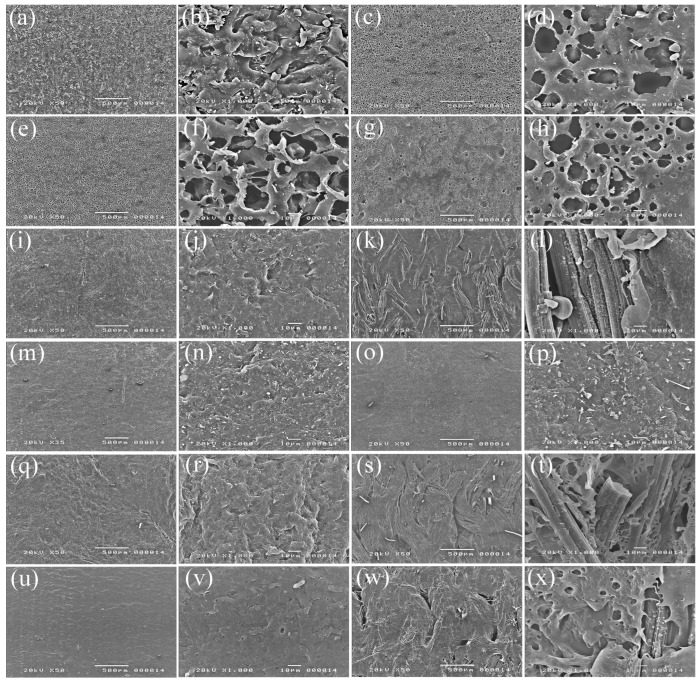
SEM images showing the microstructure of fabricated cefazolin loaded samples: (**a**) PCL_25 at 50×; (**b**) PCL_25 at 1000×; (**c**) PCL_50 at 50×; (**d**) PCL_50 at 1000×; (**e**) PCL_75 at 50×; (**f**) PCL_75 at 1000×; (**g**) PCL_100 at 50×; (**h**) PCL_100 at 1000×; (**i**) P20_25 smooth side at 50×; (**j**) P20_25 smooth side at 1000×; (**k**) P20_25 rough side at 50×; (**l**) P20_25 rough side at 1000×; (**m**) P20_50 smooth side at 50×; (**n**) P20_50 smooth side at 1000×; (**o**) P20_50 rough side at 50×; (**p**) P20_50 rough side at 1000×; (**q**) P20_75 smooth side at 50×; (**r**) P20_75 smooth side at 1000×; (**s**) P20_75 rough side at 50×; (**t**) P20_75 rough side at 1000×; (**u**) P20_100 smooth side at 50×; (**v**) P20_100 smooth side at 1000×; (**w**) P20_100 rough side at 50×; (**x**) P20_100 rough side at 1000×. Magnification 50× (bar = 500 µm) and magnification 1000× (bar = 10 µm).

Figure 1 shows the microstructure of cefazolin loaded samples. Regardless of cefazolin concentrations, all cefazolin loaded PCL generally displayed an open microstructure resulting from the exchange of solvent and water in the fabrication process (Figure 1a,c,e,g). However, the amount of dense area surrounding pores tended to increase with increasing cefazolin content in the PCL solution. Upon examining higher magnification images (Figure 1b,d,f,h), only PCL_25 showed a bicontinuous-like structure having a pore size less than 5 µm while PCL_50, PCL_75 and PCL_100 showed cellular structure having larger pore size of about 10–20 µm. In contrast, cefazolin loaded ORC/PCL bilayered composites exhibited a bilayer structure comprising a nonporous layer and a composite layer. In the case of the nonporous layer side, all formulations similarly displayed dense solid structure of PCL similar to those of cefazolin loaded PCL, but no pores were observed in this case (Figure 1i,j,m,n,q,r,u,v). In the case of the composite layer side, different microstructures were obtained depending on the cefazolin concentrations. At a low cefazolin concentration of 25 mg/mL, the composite layer side consisted of knitted ORC fabric mostly embedded within the continuous PCL matrix with exposed fabric in some areas (Figure 1k). Increasing the cefazolin concentration to 50 mg/mL resulted in a dense PCL layer without any exposed ORC fabric resembling the dense structure of the nonporous layer side (Figure 1o). Further increasing the cefazolin concentration to 75 and 100 mg/mL led to the coating of PCL on top of the ORC fabric since the knitted ORC morphology was still evidenced, but with the thick coating of the PCL layer on top (Figure 1s,w). At high magnification, PCL matrix was observed to be dense for P20_25 and P20_50 (Figure 1l,p) while some pores were observed in the PCL matrix close to the ORC fibers for P20_75 and P20_100 (Figure 1t,x).

**Figure 2:**
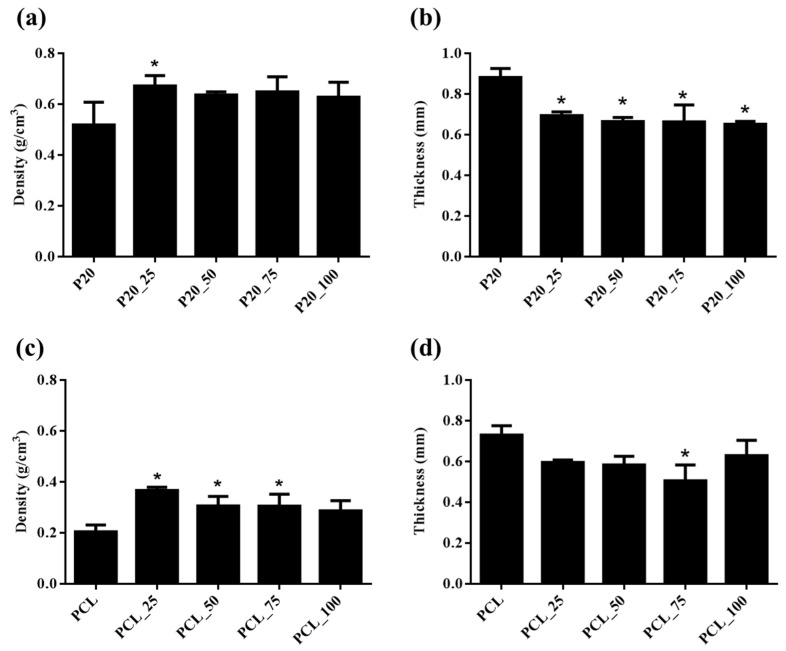
Influence of cefazolin concentration in the ORC/PCL bilayered composite and PCL on density and thickness of fabricated samples: (**a**) Density of cefazolin loaded ORC/PCL bilayered composite; (**b**) thickness of cefazolin loaded ORC/PCL bilayered composite; (**c**) density of cefazolin loaded PCL; (**d**) thickness of cefazolin loaded PCL. Data are expressed as mean ± standard deviation (SD). Significance between cefazolin loaded samples versus control (P20 or PCL) is indicated as * (*p* < 0.05).

Figure 2 shows the bulk density and thickness of cefazolin loaded and drug unloaded samples. The bulk density and thickness of all formulations of cefazolin loaded ORC/PCL bilayered composite did not differ significantly. In comparison to their corresponding drug unloaded samples (P20 or PCL), bulk density of both cefazolin loaded ORC/PCL bilayered composite and cefazolin loaded PCL samples was greater, but the thickness was lower. However, the significant difference (*p* < 0.05) in bulk density between cefazolin loaded and drug unloaded samples was seen for P20_25 and all cefazolin loaded PCL except PCL_100. In contrast, thickness of all cefazolin loaded ORC/PCL bilayered composites was significantly lower than that of P20 while only that of PCL75 was significantly different. 

#### 3.1.2. Tensile Properties

**Figure 3:**
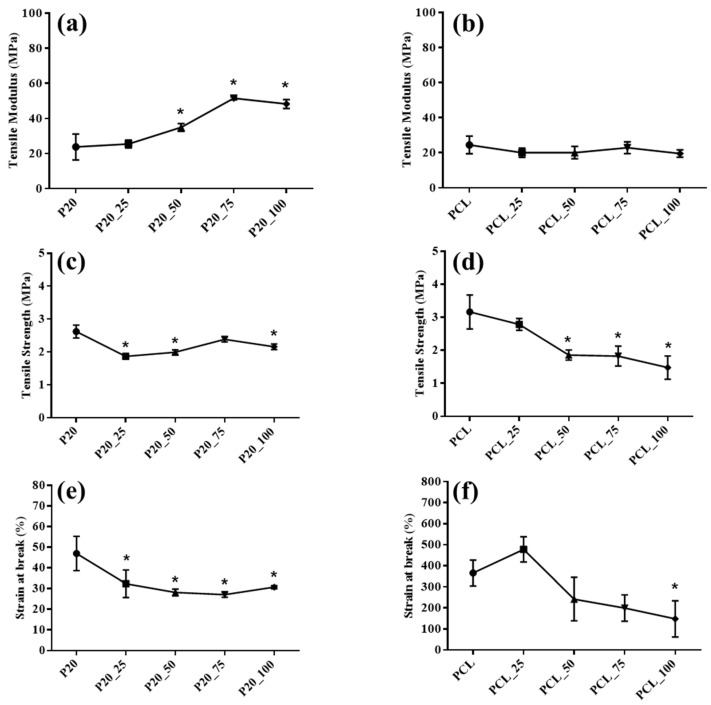
Influence of cefazolin concentration in the ORC/PCL bilayered composite and PCL on tensile properties: (**a**) Tensile modulus of cefazolin loaded ORC/PCL bilayered composite; (**b**) tensile modulus of cefazolin loaded PCL; (**c**) tensile strength of cefazolin loaded ORC/PCL bilayered composite; (**d**) tensile strength of cefazolin loaded PCL; (**e**) tensile strain at break of cefazolin loaded ORC/PCL bilayered composite; (**f**) tensile strain at break of cefazolin loaded PCL. Data are expressed as mean ± standard deviation (SD). Statistical significance between cefazolin loaded samples versus control (P20 or PCL) is indicated as * (*p* < 0.05).

Figure 3 shows tensile properties of drug loaded and unloaded samples. Tensile modulus of cefazolin loaded ORC/PCL bilayered composite increased with increasing cefazolin concentration, which reached statistical significance when cefazolin concentration was 50 mg/mL or above (Figure 3a). However, this was not observed in cefazolin loaded PCL, in which the tensile moduli of all samples were seen to be in the same range and did not differ significantly from that of PCL (Figure 3b). Generally, tensile strengths of both cefazolin loaded ORC/PCL bilayered composite and cefazolin loaded PCL were found to be significantly lower compared to unloaded samples (*p* < 0.05). Tensile strength of cefazolin loaded PCL samples tended to decrease when cefazolin concentration was increased while those of cefazolin loaded ORC/PCL bilayered composite remained relatively unchanged (Figure 3c,d). All cefazolin loaded ORC/PCL bilayered composites exhibited similar tensile strain at break regardless of the concentration of cefazolin, but all were significantly lower than that of drug unloaded composite (Figure 3e). In contrast to cefazolin loaded ORC/PCL bilayered composite, tensile strain at break of cefazolin loaded PCL samples tended to decrease with increasing cefazolin concentration, but only reached statistical significance at the highest concentration of cefazolin (*p* < 0.05) (Figure 3f). Comparing between cefazolin loaded ORC/PCL bilayered composite and cefazolin loaded PCL at a similar cefazolin concentration, the composites had greater tensile modulus than those of PCL, but tensile strengths were in similar ranges. Tensile strain at break of cefazolin loaded PCL was much greater than those of cefazolin loaded ORC/PCL bilayered composite.

### 3.2. Total Cefazolin Content 

The measured total cefazolin loading contents in fabricated cefazolin loaded ORC/PCL bilayered composite and cefazolin loaded PCL are shown in Table 2. Increasing cefazolin concentration in the solution significantly increased the uptake of cefazolin in both types of fabricated samples. For a similar cefazolin concentration employed, the loading content of cefazolin in the ORC/PCL bilayered composite was greater than that of cefazolin loaded PCL. 

### 3.3. In Vitro Cefazolin Release 

**Figure 4:**
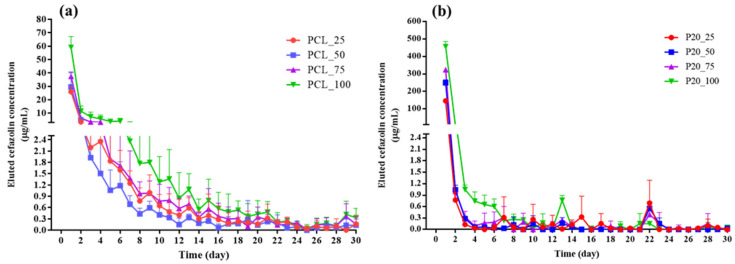
Cefazoline release profile of cefazolin loaded samples in aCSF solution: (**a**) Cefazolin loaded PCL; (**b**) cefazolin loaded ORC/PCL bilayered composites. Data are expressed as mean ± standard deviation (SD).

**Figure 5:**
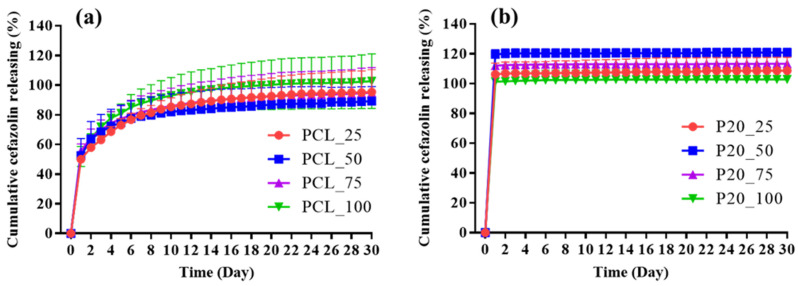
Cumulative cefazoline release profile of cefazolin loaded samples in aCSF solution: (**a**) Cefazolin loaded PCL; (**b**) cefazolin loaded ORC/PCL bilayered composites. Data are expressed as mean ± standard deviation (SD).

Figure 4 shows the daily concentration of eluted cefazolin (μg/mL) from cefazolin loaded samples for up to 30 days. All samples displayed similar release profiles including a sharp initial burst release of the highest concentration of cefazolin on the first day, followed by a significantly declined release thereafter. While cefazolin was gradually eluted from cefazolin loaded PCL samples for approximately 15 days (Figure 4a), almost all cefazolin was rapidly eluted from cefazolin loaded ORC/PCL bilayered composite during the 4–10 days depending on the cefazolin loading (Figure 4b). Figure 5 shows the cumulative percentage of released cefazolin as a function of time for cefazolin loaded samples over 30 days. The burst and delayed release were observed for cefazolin loaded PCL samples (Figure 5a), where the cumulative percentage of released cefazolin was found to reach approximately 50% in 1 day. In contrast, cefazolin loaded ORC/PCL bilayered composite exhibited a greater burst release, in which most of cefazolin was released in 1 day and followed by constant small release thereafter (Figure 5b). No differences in daily released content or cumulative release among different formulations for both cefazolin loaded PCL and cefazolin loaded ORC/PCL composite were observed.

### 3.4. Minimum Inhibitory Concentration (MIC)

Table 3 shows a series of half-decreasing concentrations of cefazolin employed against *S. aureus* ATCC 50923. Based on this result, the MIC value, the lowest concentration of cefazolin that inhibited the bacterial growth, was 0.3150 μg/mL (Well 5).

### 3.5. Antibacterial Activity 

**Figure 6:**
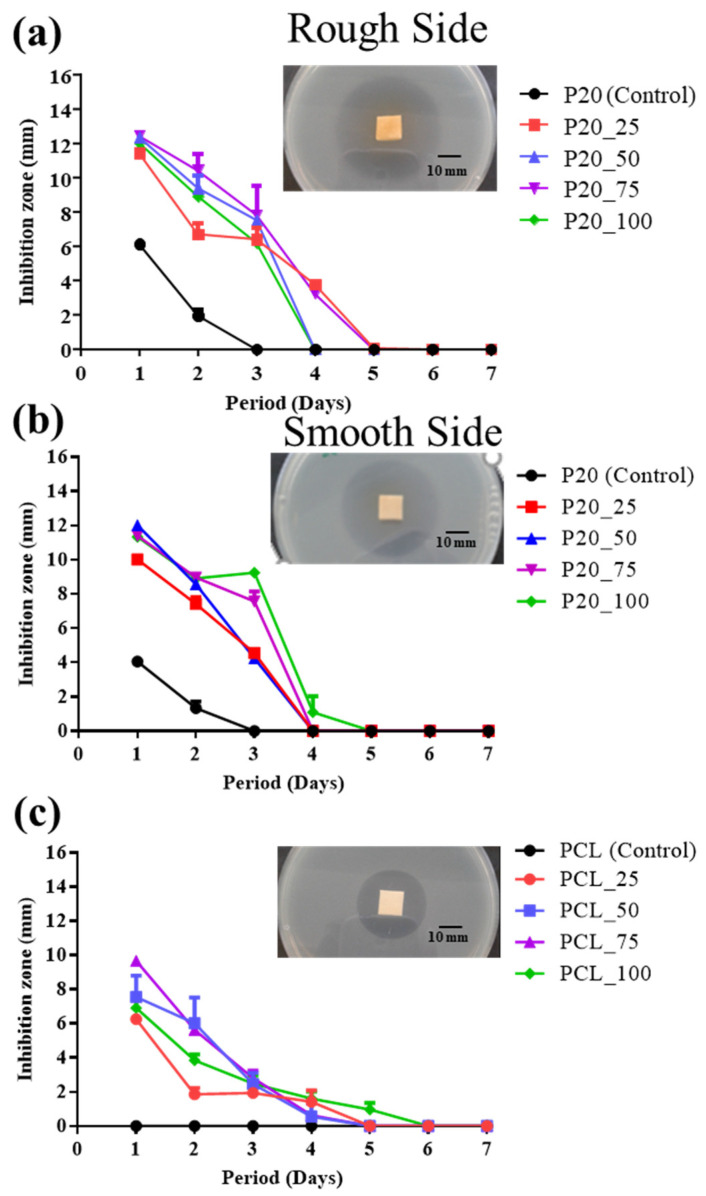
Antibacterial activity of cefazolin loaded samples against *S. aureus* as indicated by inhibition zone: (**a**) Rough side of cefazolin loaded ORC/PCL bilayered composite; (**b**) smooth side of cefazolin loaded ORC/PCL bilayered composite; (**c**) cefazolin loaded PCL. Bar = 10 mm.

Figure 6 shows the antimicrobial activity profiles of cefazolin loaded ORC/PCL bilayered composite and cefazolin loaded PCL, which were assessed by using Kirby–Bauer methods for up to 7 days. Cefazolin loaded ORC/PCL bilayered composite showed an antibacterial activity against *S. aureus* for up to 4 days while cefazolin loaded PCL showed a slightly longer activity for up to 5 days. The greatest inhibition zone was observed on day 1, which was relatively similar in both cefazolin loaded ORC/PCL bilayered composite and cefazolin loaded PCL regardless of the concentration of cefazolin. It can be noted that the inhibition zone of cefazolin loaded ORC/PCL bilayered composite tended to display a greater value compared to that of the cefazolin loaded PCL at the same concentration of cefazolin. The inhibition zone of the smooth side of the cefazolin loaded ORC/PCL bilayered composite and cefazolin loaded PCL tended to increase with increasing cefazolin content and the longest inhibition durations (4 and 5 days, respectively) were similarly found at 100 mg/mL (Figure 6b,c). Contrarily, the inhibition zone and antibacterial duration of the rough side of cefazolin loaded ORC/PCL bilayered composite did not correspond with the concentration of cefazolin loading (Figure 6a). In a similar tested period, P20_75 tended to show the greatest inhibition zone while P20_100 showed the second smallest inhibition zone. In the case of antibacterial duration, P20_50 and P20_75 displayed a longer period than P20_50 and P20_100. A drug unloaded composite (P20) also exhibited an antimicrobial activity on day 1 which decreased thereafter, in which the rough side (Figure 6a) exhibited a greater inhibition zone (6.1 ± 0.1 mm) compared to the smooth side (4.1 ± 0.2 mm) (Figure 6b). However, the inhibition zones of P20 on both sides were still smaller than those of cefazolin loaded ORC/PCL bilayered composite. In contrast, the drug unloaded PCL did not show any antibacterial activity. 

## 4. Discussion

The microstructure of cefazolin loaded PCL resembled the microstructure of porous polymers that were fabricated by a nonsolvent induced phase inversion process wherein the delayed demixing took place when the NMP exchanged into water and precipitation occurred in the PCL solution [30,31]. The microstructure was also observed to be influenced by the cefazolin content which led to an increase in observed viscosity of the PCL solution similar to the increase in polymer content or additives. This caused the insufficiency solvent and nonsolvent exchange to form pores during phase separation and solidification which tended to decrease the porosity of the samples [30,31]. Pore morphology was also affected by cefazolin content. It was previously reported that several pore structures could be obtained by an immersion precipitation process including unconnected latex, nodules, bicontinuous structures, cellular structures or macrovoids depending on the parameters employed in the process, including, but not limit to, solvent and nonsolvent used, polymer solution composition and casting conditions [31,32]. In this study, a bicontinuous-like structure containing interconnected pores was attained at the lowest cefazolin concentration (PCL_25) while cellular structure resulted when using a greater cefazolin concentration. 

All cefazolin loaded ORC/PCL bilayered composites retained a bilayered structure which consisted of a composite layer and nonporous PCL layer similarly to those of drug unloaded ORC/PCL bilayered composite, P20 [3]. However, the microstructure of the composite side resembled that of P20 only at the lowest cefazolin loading (P20_25) wherein the viscosity of PCL solution was still comparable and could infiltrate the ORC fabric readily, but not fully cover the surface since the PCL content was low. Using the immediate viscosity solution of P20_50, a fraction of the solution would still be able to infiltrate and fill the gaps among the fibers of ORC and the surplus part that could not infiltrate would spread and coat on the surface instead which resulted in the smooth coating on the surface. Using high viscosity solution of P20_75 and P20_100, limited infiltration was achieved and the majority of the solution would coat on the ORC while preserving the ORC morphology underneath. The coating layer of P20_100 was observed to be thicker than that of P20_75. These changes in solution viscosity and microstructure with increasing cefazolin content were in the same fashion as increasing PCL concentration in fabricating ORC/PCL composite as reported previously [3]. Unlike cefazolin loaded PCL, no effect of such an increase in viscosity was noted on the dense PCL side and all formulations similarly displayed nonporous structure. This was thought to be partly due to the solvent absorption by ORC fabric which concentrated the PCL solution and the easier transport of the solvent though the composite side. These could depress the exchange of solvent and nonsolvent at the dense PCL side, resulting in demixing without pore generation. 

Cefazolin loaded ORC/PCL bilayered composite exhibited greater densities, but lower thickness, than drug unloaded composite. These changes after cefazolin loading were also observed in cefazolin loaded PCL. These could all be related to the increase in solution viscosity which decreased the swelling ability of the samples during solvent–water exchange in the solidification step and the decrease in coating layer due to the difficulty to achieve the spreading during the infiltration and recoating process. Despite this, the thickness (0.65–0.69 mm) of the composites was still in the same range as those observed in human dura (0.3–0.8 mm) [33], while densities (0.63–0.67 g/cm^3^) were lower than that of human dura (1.03 g/cm^3^) [34]. These results suggested that cefazolin loaded composites could still be employed and handled similarly to the drug unloaded ORC/PCL bilayered composite and natural dura mater.

Apart from physical properties, changes in tensile properties were also observed after cefazolin loading. Cefazolin loaded PCL was weaker and less ductile after cefazolin loading since the tensile strength and strain at break decreased with increasing cefazolin concentration, while the tensile modulus was relatively unchanged (Figure 3b,d,f). This was consistent with the previous findings for other types of drug loaded PCL [35,36,37,38]. In contrast, tensile strength of cefazolin loaded ORC/PCL bilayered composite was unaffected, but the increase in tensile modulus and the decrease in strain at break with increasing cefazolin concentration were observed instead (Figure 3a,e). Therefore, the underlying mechanism might be different. The changes in tensile properties observed were anticipated to be due to the effect of incorporated cefazolin that could restrict both the deformation of ORC fabric as well as the polymer chain movement of the PCL matrix. In the case of cefazolin loaded PCL, the restriction of PCL chain movement imposed by cefazolin obviously increased the brittleness and decreased the strength of the sample. In the case of ORC/PCL bilayered composite, the reinforcing effect of ORC fabric was relatively greater and might have offset the effect of cefazolin on strength reduction due to PCL chain movement restriction resulting in overall unchanged tensile strength. The restraint of ORC deformation imposed by cefazolin also amplified the stiffness of ORC and gave rise to the increase in tensile modulus. Despite the changes in the tensile properties of all cefazolin loaded ORC/PCL bilayered composites, these tensile values were still within the same range of human dura mater as reported previously, including 11.2–171.5 MPa, 1.3–27.1 MPa and 16.0–49.7% for tensile modulus, tensile strength and strain at break, respectively [34,39,40].

The release profiles of cefazolin loaded PCL were biphasic, comprising the burst release of approximately half of the total cefazolin content and followed by a gradual release for 15 days which was similar to typical burst biphasic release kinetics of drug loaded polymer matrix wherein the initial burst release phase is followed by a power-law phase resulting from Fickian diffusion processes or “anomalous” processes which encompass both diffusion of drugs and swelling of polymers [41,42]. In contrast, the release profile of cefazolin loaded ORC/PCL bilayered composite was found to be mainly burst release where most of the cefazolin was eluted in this phase and followed by relatively constant release thereafter for up to 4–10 days. This is monophasic release kinetics comprising a zero-order release phase that displays a constant rate of drug release via non-Fickian diffusion [42]. The difference in these release profiles between two types of cefazolin loaded samples could be related to the dissimilarity of the composition and microstructure as observed. The cefazolin loaded ORC/PCL bilayered composite contained two layers comprising a composite layer which in situ produced porous channels by relatively faster ORC resorption and the relatively slower degradation of the nonporous PCL layer. These microstructures would aid the diffusion of aCSF solution into the samples, mainly through the composite layer, and caused a rapid dissolution and release of cefazolin resulting in the burst release as observed. Since three different mechanisms of drug release from the polymer matrix were reported, including release of surface loaded drug, diffusion and degradation of the carrier [43,44], the release of antibiotics from dense PCL layer in ORC/PCL bilayered composite could also occur, but contributed less than that of the composite layer. In the case of cefazolin loaded PCL, the diffusion pathway of aCSF into the PCL matrix to dissolve and lead to the release of cefazolin would be more difficult and drug would be slowly released [45]. The initial rapid release of cefazolin loaded PCL might be due to the release of surface loaded cefazolin and part of the drugs which were close to the inner surface of PCL. A subsequent gradual release would be caused by the diffusion and swelling or degradation of PCL. Moreover, both release phases of cefazolin loaded PCL would also be amplified by the open porous microstructure which could act as an additional route for solution diffusion and drug release. Compared to other cefazolin loaded carriers [20,21,22,23,24,25,26], cefazolin loaded PCL fabricated in this study exhibited similar biphasic release kinetics to those reported previously, especially when using PCL as a carrier. The rate of elution tended to be faster than that of dense electrospun fiber [21], but comparable to that of PCL sponge which contained microscopic pores [20]. In contrast, ORC/PCL bilayered composite displayed much faster release of cefazolin than cefazolin loaded carriers reported previously and also exhibited different release kinetics. It should be noted that the limitation of using of a UV–Vis spectrophotometer for cefazolin measurement is that it might not specifically detect the cefazolin compared to chromatographic or mass spectrometric methods. 

Concerning the MIC of cefazolin against *S. aureus* which was determined to be 0.3150 μg/mL, the duration of the release content of cefazolin from cefazolin loaded ORC/PCL bilayered composite which was still closed to MIC value was approximately 3 days for all samples, except that of P20_100, which was 10 days. This correlated quite well with the results of antibacterial activity which was found to last for 3–4 days. In the case of cefazolin loaded PCL, the duration for which the released cefazolin concentration was still in MIC range was about 14 days which differed from the antibacterial activity durations of 4–5 days. This could be due to the dissimilarity in the solution saturated condition in the release study compared to the moistened agar in antibacterial activity testing which would affect the cefazolin release, in particular from PCL matrix. Interestingly, it was also observed that a drug unloaded ORC/PCL composite (P20) also exhibited antibacterial activity. This was probably due to the known antibacterial property of ORC that was reported to produce an acidic environment which was not suitable for microorganisms to survive [46,47]. The antibacterial activity of ORC in the composites can be further confirmed by observation that a greater inhibition zone was observed from the rough side of the composite where the knitted ORC fabric was presented compared to that of the smooth PCL side. However, no clear difference in the inhibition zone of the cefazolin loaded ORC/PCL composite between the rough side and smooth side was observed. This was possibly because the antibacterial activity of released cefazolin was much greater than that of ORC and dominated the activity. In addition, the greater transport ability of solution through the rough side might not be advantageous over the smooth side in this contact situation compared to full immersion in the release test.

The burst effect can be regarded as a negative consequence when long-term controlled release applications are needed, but a rapid release or high initial rates of delivery may be the optimal mechanism in certain situations [48]. It should be noted that the purpose of antimicrobial prophylaxis is to achieve drug levels in serum and tissue that surpass the MIC of the bacterial organisms likely to be faced during operation and prophylaxis after wound close is unessential [49,50,51]. Therefore, the burst release of cefazolin loaded ORC/PCL bilayered composite, which could be a problem in some applications which require a sustained release of drug for a long period of time, might not be relevant for an antibacterial dural substitute, which requires a localized release of high concentration of antibiotic prophylaxis to ensure that any bacterial infections (if any) are totally and immediately eliminated at the site of implantation. Additionally, the burst release of a high concentration of cefazolin observed in the present study could be an advantage as this could reduce the risk of antibiotic resistance. 

## 5. Conclusions

The antibacterial synthetic dural substitute was successfully fabricated by incorporating cefazolin into ORC/PCL bilayered composite, which still had the physical and mechanical properties in the range of natural dura mater. Cefazolin released from ORC/PCL bilayered composite was found to be monophasic, comprising mainly a burst release. Among all formulations, P20_25 might be most suitable since it showed relatively similar releasing profile and antibacterial activity to other higher drug loaded formulations and still exhibited comparable microstructure and physical and mechanical properties to the drug unloaded composite. Although cefazolin loaded ORC/PCL bilayered composite was found to exhibit a burst release of antibiotic, in which its antibacterial activity was sustained for up to 4 days, this might not fully predict in vivo performance where the environment and the clearance of the released cefazolin from the site of injury might be different. Further studies in animals are required to confirm its performance prior to translating to clinical study.

## Figures and Tables

**Table 1:** Formulations of the prepared samples.

Samples	Cefazolin (g)	NMP (mL)	PCL (g)	ORC Impregnation
P20	0	100	20	Yes
P20_25	2.5	100	20	Yes
P20_50	5.0	100	20	Yes
P20_75	7.5	100	20	Yes
P20_100	10.0	100	20	Yes
PCL	0	100	20	No
PCL_25	2.5	100	20	No
PCL_50	5.0	100	20	No
PCL_75	7.5	100	20	No
PCL_100	10.0	100	20	No

**Table 2:** Total cefazolin contents loaded in the samples. Data are expressed as mean ± standard deviation (SD).

Samples	Total Cefazolin Content (mg Drug per 100 mg Sample)
P20_25	1.94 ± 0.15
P20_50	2.77 ± 0.17
P20_75	3.61 ± 0.07
P20_100	5.54 ± 0.09
PCL_25	1.74 ± 0.10
PCL-50	2.49 ± 0.13
PCL_75	2.80 ± 0.11
PCL_100	3.28 ± 0.11

**Table 3:** Minimum inhibitory concentration (MIC) value of cefazolin against *S. aureus* ATCC 50923.

Well Number	Cefazolin Concentration (μg/mL)	Bacterial Growth (х = No Growth; Clear Solution, ✓= Growth; Turbid Solution)
1	5.0	х
2	2.5	х
3	1.50	х
4	0.650	х
5	0.3150	х
6	0.1563	✓
7	0.0780	✓
8	0.0390	✓
9	0.0195	✓
10	0.0	✓
11	Nutrient broth (NB), no bacterial inoculum	х
12	NB + aCSF, no bacterial inoculum	х

## Data Availability

The data presented in this study are available on request from the corresponding author. The data are not publicly available due to confidentiality.
